# Challenging Diagnosis of Anomalous Origin of the Right Coronary Artery from the Pulmonary Artery

**DOI:** 10.3390/diagnostics12112671

**Published:** 2022-11-03

**Authors:** Hiyam Mahmoud, Eliza Cinteză, Cristiana Voicu, Irina Mărgărint, Iulian Rotaru, Amelia Aria, Tammam Youssef, Alin Nicolescu

**Affiliations:** 1 “Marie Curie” Emergency Children’s Hospital, 041451 Bucharest, Romania; 2 Royal Brompton Hospital, London SW3 6NP, UK; 3 Department of Pediatrics, Faculty of Medicine, “Carol Davila” University of Medicine and Pharmacy, 020021 Bucharest, Romania

**Keywords:** coronary artery disease, ARCAPA, congenital heart disease, reimplantation, ischemia, coronary angiography

## Abstract

Anomalous origin of the right coronary artery (RCA) from the pulmonary artery, ARCAPA, is an extremely rare congenital heart disease. Only 200 cases were reported from 1885 to the present. Patients diagnosed with ARCAPA can be either asymptomatic or can experience symptoms, such as heart murmur, dyspnea, or angina, shortly after birth or around 40–60 years of life. Usually, those with isolated ARCAPA are diagnosed later in life compared to those who associate other structural cardiac defects. We report two cases of anomalous origin of the right coronary artery at the level of the pulmonary artery trunk (ARCAPA) that were diagnosed by invasive coronary angiography. Although asymptomatic, general recommendations suggest an early corrective intervention to prevent complications such as myocardial ischemia and cardiac dysfunction, which can lead to sudden cardiac death.

## 1. Introduction

An anomalous right coronary artery from the pulmonary artery (ARCAPA) is one of the rarest congenital coronary anomalies and is found in approximately 0.002% of the general population [1,2]. Brooks described this anomaly for the first time in 1885. When not combined with other congenital heart defects, this condition is often asymptomatic and is found incidentally. There is a significant difference compared to the anomalous origin of the left coronary artery from the pulmonary artery (ALCAPA), considered the most common type of coronary artery origination anomaly from the pulmonary artery, especially regarding the high rate of mortality in infancy and early childhood [1,2,3,4,5,6,7]. However, some patients with ARCAPA also may suffer from exertion angina, and cases of sudden cardiac death were reported as well [8]. When the right coronary artery (RCA) originates from the pulmonary artery, “steal syndrome” may cause exercise-induced myocardial ischemia. This may be the mechanism of sudden death, and if the diagnosis is confirmed, then surgical correction appears warranted. We report two pediatric cases of ARCAPA diagnosed over the last year at the “Marie Curie” Emergency Children’s Hospital in Bucharest, Romania, and their postoperative evolution after the surgical correction of the coronary anomaly.

## 2. Case Report #1

A 3-year-old girl was admitted for a complete evaluation suspected of having a coronary fistula since the age of 2 months. Although asymptomatic with a normal clinical evaluation, the patient presented progressive dilation of the left coronary artery during regular cardiac echocardiographic monitoring.

**Figure 1:**
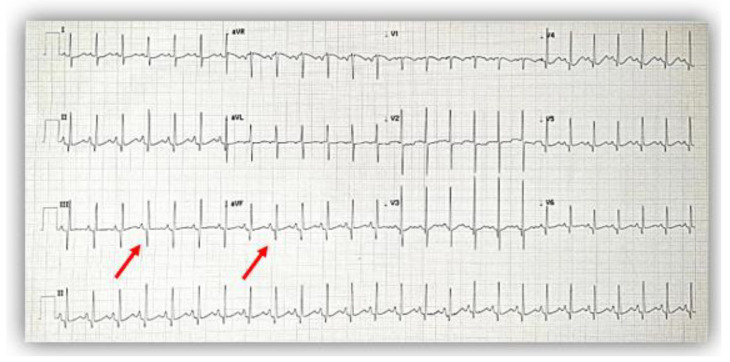
The electrocardiogram at admission showed a normal pattern except for deep Q waves (red arrows) in inferior leads (DIII, aVF).

The electrocardiogram (ECG) showed sinus rhythm; a heart rate of 115/min.; normal QRS axis; normal P, QRS, and T waves morphology, specific to age; normal PR, QRS, and QT intervals, but with deep Q waves in inferior leads (DIII, aVF) (Figure 1).

**Figure 2:**
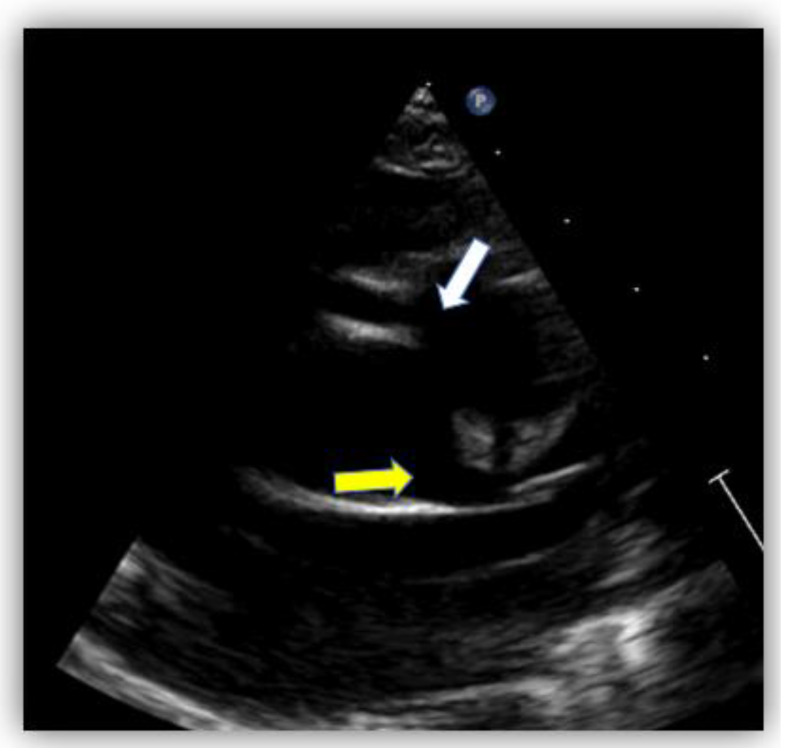
Echocardiography in the parasternal short-axis view. The white arrow shows the origin of the right coronary artery (RCA) from the pulmonary artery and the yellow arrow shows the normal origin of the left coronary artery (LCA) from the aorta.

**Figure 3:**
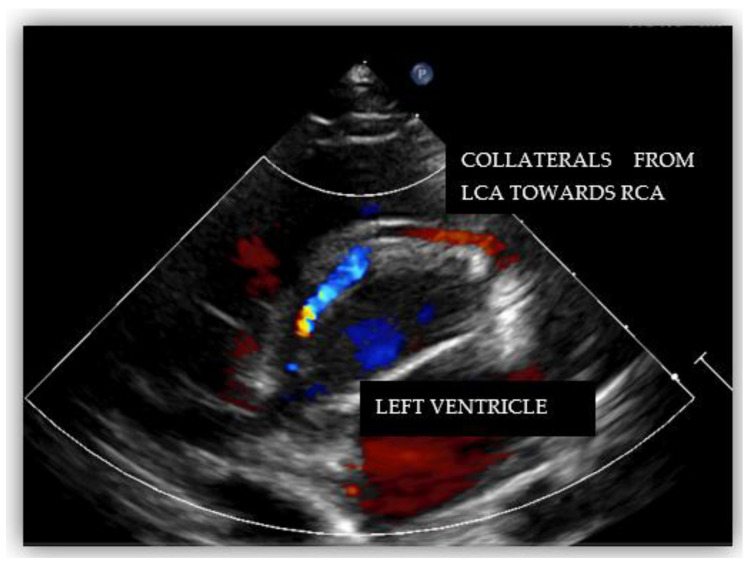
Echocardiography in parasternal short-axis showing collateral circulation.

Echocardiography showed normal structure and function of the heart. Regarding the origin of the coronary arteries, the left coronary artery (LCA) origin was revealed from the aorta, in the normal place (Figure 2). Regarding the right coronary artery, this originated apparently from the pulmonary artery, with doubt from some echocardiographic images. Having this doubt, an invasive coronary angiography was mandatory for a positive diagnosis. From multiple windows, a dilated collateral system was evident (Figure 3).

**Figure 4:**
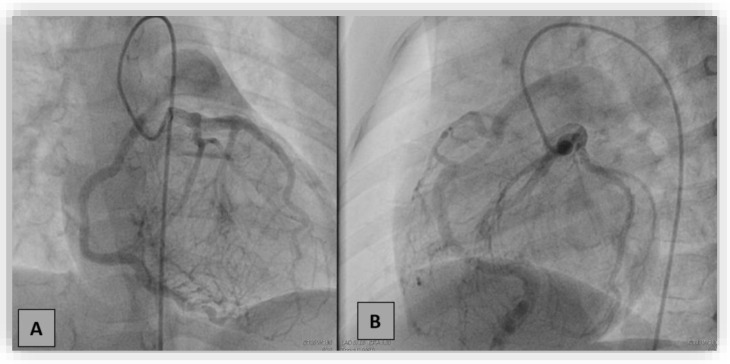
Coronarography in the right anterior oblique with cranial angulation (**A**) and left lateral oblique with caudal angulation (**B**) projections. Selective injection in the left coronary artery showed dilated left coronary artery and branches with a dense collateral network directed towards the right coronary artery (RCA), retrogradely filling RCA and the pulmonary artery.

Coronarography showed an increased diameter of the left main coronary artery up to 4 mm, extensive collateral circulation between the left and right coronary arteries, and retrograde filling of the right coronary towards the pulmonary artery (the “stilling effect”) (Figure 4). Furthermore, we performed contrast angiography of the pulmonary artery, revealing the origin of the enlarged RCA (6.9 mm) at the level of the pulmonary artery trunk (ARCAPA).

We referred the patient to cardiac surgery, where the right coronary artery was translocated from the pulmonary artery to the ascending aorta with assisted extracorporeal circulation on the beating heart. The right coronary artery mouth was ligated, cut, and reimplanted into the anterior ascending aorta, where the aorta was clamped partially with a Satinsky clamp.

**Figure 5:**
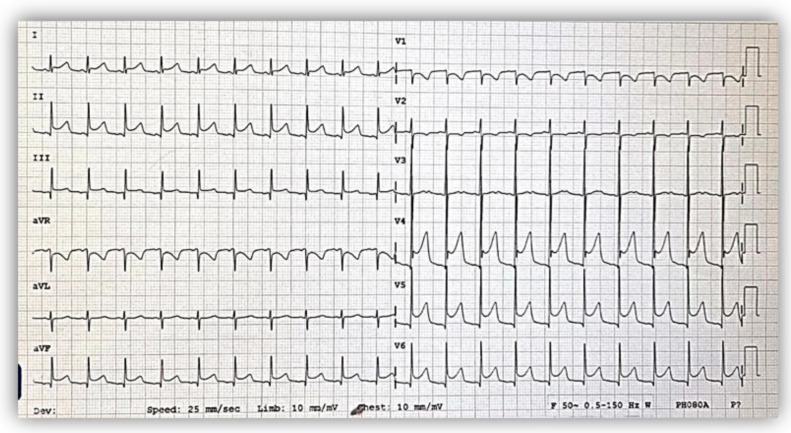
Postoperative electrocardiogram showing ST elevation in DII, DIII, aVF, and V4-V6 derivations, suggesting flow disturbance in the right coronary artery territory.

Although the echocardiographic postoperative assessment showed normal implantation of the RCA with good blood flow in both coronary arteries and no obstructions, a few hours after the surgery, the patient experienced an ischemic phenomenon: ST-segment elevation in DII, DIII, aVF, and V4-V6 derivations (Figure 5) with right ventricle moderate-severe hypokinesia with an estimated ejection fraction of 30% and positive Troponin T, 85 ng/L (normal value 14 ng/L). The most probable etiology was transient coronary spasm due to the change from low to high pressure, which resolved spontaneously.

At the 6-month follow-up, the patient’s clinical condition was normal and the echocardiography indicated coronary arteries of normal size.

## 3. Case Report #2

A 2-year-10-month-old male child diagnosed in-utero with an aortopulmonary window who beneficiated from a surgical correction immediately after birth was admitted with suspicion of a coronary anomaly.

After the cardiac surgery in the neonatal period for the aortopulmonary window, the patient was closely monitored because echocardiographic reevaluation raised the suspicion of a coronary artery anomaly.

**Figure 6:**
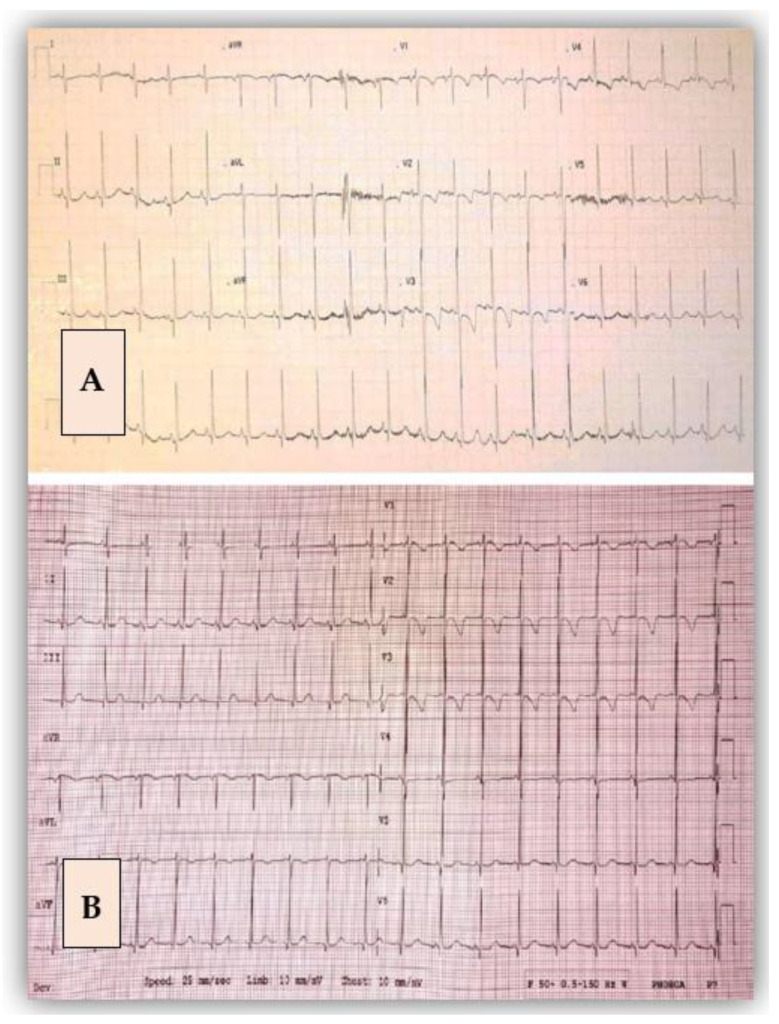
Electrocardiogram before (**A**) and after the surgical intervention (**B**). After the surgery, there was a flat T wave in V4 compared with the initial image.

Clinical examination was normal for his age, except for a central scar at the level of the thorax and a second-degree II/VI systolic murmur. The electrocardiogram revealed right axis deviation (100°), and a negative T wave extended from V1 up to V4 (Figure 6A).

**Figure 7:**
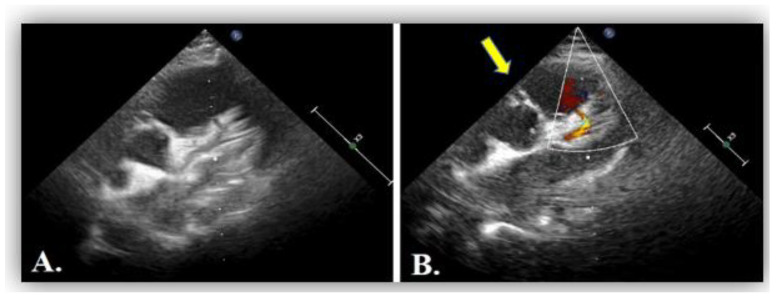
(**A**) Transthoracic echocardiography in parasternal short axis view showing dilated left coronary artery, LCA. (**B**) Artefactuous image of the right coronary artery showing the apparently normal origin of the right coronary artery from the aorta at the 11 o’clock position (yellow arrow).

**Figure 8:**
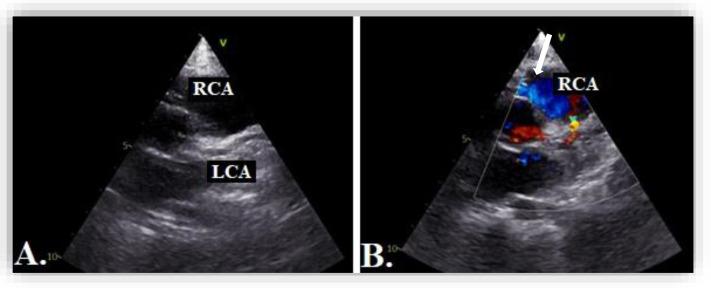
Transthoracic echocardiography in parasternal short axis view showing 2D image (**A**) and color Doppler imaging (**B**) of the right coronary artery, RCA (blue flow, white arrow).

The echocardiographic assessment showed normal structural and functional evaluation, with good contractility and normal cardiac chamber dimensions. Coronary artery dilatation was noticed, with the left main coronary artery measuring 3.7 mm (Montreal Z score = +3.55) (Figure 7A,B). The right coronary artery origin was shifted to the left (around the 2 o’clock position). At this level, a diastolic flow towards the pulmonary artery was noticed and regarded as a “vascular steal” phenomenon, which raised the suspicion of an anomalous origin of the right coronary artery. The right coronary artery appeared to be implanted in the pulmonary artery (Figure 8), but misleading images showed apparently normal insertion of the RCS origin into the aorta (see yellow arrow in Figure 7B). Furthermore, several fistulas between the coronaries and the right ventricle were depicted.

**Figure 9:**
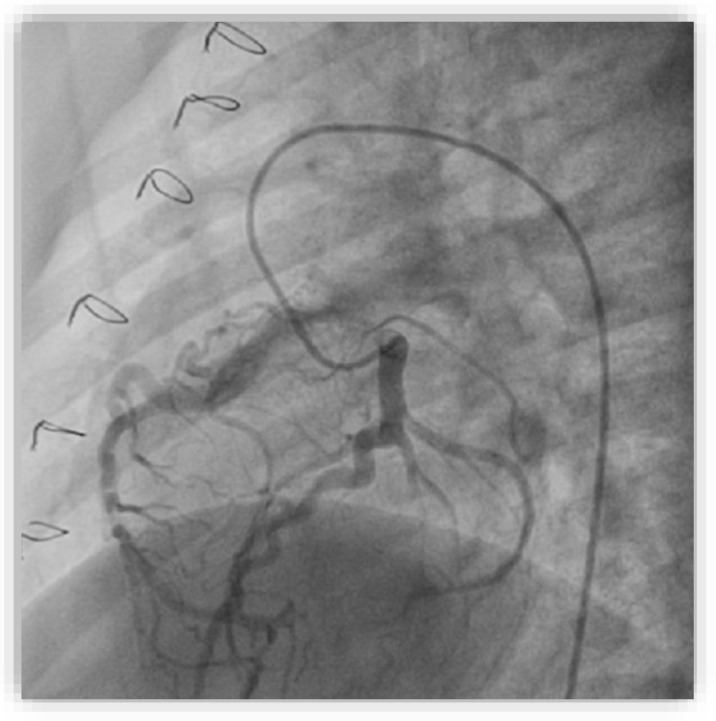
Selective coronary angiography into the left coronary artery in the anteroposterior view with left and caudal angulation showing dilated left coronary arteries and branches with collateral circulation towards the right coronary artery connected to the pulmonary artery.

We performed diagnostic catheterization and coronary angiography that confirmed the increased diameter of the LCA and its branches, with collateral circulation towards the RCA (Figure 9). The RCA was connected to the pulmonary artery. A PVR study indicated 0.6 WU and Qp/Qs ratio was 3.25.

Our patient was scheduled one month later for cardiac surgery. The ostium of the RCA was detached from the pulmonary artery and reimplanted into the aorta. The pulmonary artery was enlarged using bovine pericardium. Weaning off the cardio-pulmonary bypass was performed uneventfully.

This patient had a good post-operative evolution with no complications, and he was discharged one week after the surgery in good clinical condition. At a three-month follow-up, the evolution was favorable.

## 4. Discussion

Anomalous origin of the coronary arteries arising from the pulmonary artery (AOCAPA) is a very rare entity. The most common type is the anomalous origin of the left main coronary artery from the pulmonary artery, also known as ALCAPA. The right coronary arising from the pulmonary artery accounts for just 10–20% of all AOCAPA. Furthermore, other types were reported, such as a left anterior descending artery or circumflex branch anomalously arising from the pulmonary artery, both coronary arteries from the PA known as total anomalous origin, or the left main coronary from the right pulmonary artery [9].

Cleveland Clinic Foundation performed one study on 126,595 patients who underwent coronary angiography over three decades (1960–1988) and concluded that isolated coronary anomalies occur in 1.3% of patients [5]. Similar results were obtained in another study performed on 12,457 adult patients using coronary angiography [10]. Among the patients discovered with coronary anomalies, approximately 20% were considered at high risk, having potentially serious anomalies, such as ectopic coronary origin from the pulmonary artery, ectopic coronary origin from the opposite aortic sinus, single coronary artery, and large coronary artery fistulae [5].

ARCAPAs may appear isolated or associated with other congenital heart diseases. Williams et al. conducted a review of the literature on 70 patients diagnosed with ARCAPA in published case reports and described a possible association with other structural cardiac lesions in more than one-third of the reports [3]. The most common associated lesions were: aortopulmonary window and tetralogy of Fallot, but additional lesions were encountered: ventricular septal defect, atrial septal defect, double outlet right ventricle, patent ductus arteriosus, coarctation of the aorta, aortic arch hypoplasia, pulmonary stenosis, aortic stenosis, and left main coronary artery-to-pulmonary artery fistula [3,11,12].

Because of high intramural pressure in systole, the normal physiology of blood flow through the coronary arteries is during diastole. According to this principle, the coronary perfusion pressure depends on the great artery’s diastolic pressure, which is connected to, in this case, the pulmonary artery itself. During fetal life and immediately after birth, aortic and pulmonary pressure are similar; therefore, there is no difference in coronary artery rheology. As soon as the pulmonary vascular resistance and pulmonary artery pressure drop, the coronary connected to the PA is hypoperfused (right coronary artery). As a compensatory mechanism, collaterals between LCA and RCA develop. Now, due to higher pressure in the coronary capillary bed than in the pulmonary artery, blood will flow from the LCA through the collaterals into the RCA and go back into the PA. When these collaterals are well developed, the aorta through the LCA provides sufficient perfusion to supply the entire myocardium, and as a consequence, the LCA will dilate, functioning as a left-to-right shunt. Otherwise, minimal collateral circulation will result in a coronary “steal” phenomenon that can cause ischemia and ventricular dysfunction [13].

According to the pathophysiology, the age of the presentation has a bimodal distribution with one peak near after birth (insufficient collateral circulation) and another around 40 to 60 years of age (good collaterals) [7,14]. A review conducted in 2019 has identified a total of 223 cases in 193 case reports and the median age at presentation was 14.0 years old [14].

A positive diagnosis is done starting from clinical bases and following specific investigations to confirm the abnormal origin (Table 1). Although most patients diagnosed with ARCAPA are asymptomatic, cardiac syncope, congestive heart failure, and sudden cardiac death were reported in several patients [5,15,16,17].

Our two patients were asymptomatic, both cases being diagnosed in early childhood by routine echocardiography or by post-operative follow-up echocardiography.

Transthoracic echocardiography can raise the suspicion of a coronary anomaly in 40.4% of the diagnosed cases [14]. Echocardiographic features that raise suspicion of ARCAPA are related mainly to the evaluation of the coronary arteries—the origin, proximal, and distal parts. Therefore, features such as the abnormal origin of the RCA, shifted to the left, around 2 o’clock, oriented to the pulmonary artery (PA) with the retrograde flow towards the PA, dilated left and right coronary arteries, well-developed collateral network at all levels may suggest the possibility of ARCAPA.

The progressive increase in the left coronary artery diameter with time in an otherwise healthy patient (our first case) suggested early surgical correction to prevent the risk of developing a coronary aneurysm and long-term complications.

As the literature describes, the electrocardiogram can be either normal or can indicate left ventricular hypertrophy (LVH) or deep Q waves in inferior leads [8]. Our patients did not set the voltage criteria for LVH, but the first case presented deep Q waves in leads DIII and aVF.

Diagnosis of these patients can be challenging. Usually, the dilated left coronary artery raises suspicion of the diagnosis, but color mapping of the right coronary will confirm the anomaly.

There are centers where invasive coronary diagnosis is no longer the preferred method for diagnosis, being replaced by angioCT or angioMRI [18,19,20,21,22]. Even if a correct diagnosis can be provided by angioCT or angioMRI, in adult patients, coronary angiography should be performed to have a complete diagnosis before cardiac surgery for coronary reimplantation. The adults who have a late diagnosis may add the potential for the coexistence of ischemic heart disease related to atherosclerosis, and they must be evaluated by invasive coronary angiography. A complete picture of the patient evolution up to that age can be provided by adding angioMRI for all patients, including children [23,24,25].

Although asymptomatic, general recommendations suggest an early corrective intervention to prevent complications such as myocardial ischemia and cardiac dysfunction, which can lead to sudden death [26,27,28,29]. Among the corrective interventions applied in this type of coronary anomaly, three surgical strategies are described: reimplantation of the RCA into the aorta, RCA ligation, and RCA ligation with coronary artery bypass grafting and the treatment of the associated cardiac pathology, sometimes being necessary to combine surgery with interventional cardiology for associated lesions [7,30,31].

In our cases, reimplantation of the RCA provided clinical improvement, normalization of myocardial perfusion, and prevented decreased ejection fraction and sudden death on a short-term follow-up. Only the first patient experienced a particular postoperative evolution with signs of ischemia and secondary heart failure, interpreted as a transient coronary spasm that spontaneously disappeared in a few hours.

As an algorithm, currently, the method of diagnosis is raised or made by echocardiography and confirmed by angiography [3]. Depending on the age of the child and the local possibilities of the center for evaluation and expertise, for confirmation of the diagnosis, the next step can be coronary angiography, angioCT, or angioMRI. As a surgical technique, reimplantation of the right coronary was the most common surgical procedure.

These patients, although asymptomatic, are at high risk for cardiac arrest, especially during effort. Competitive sports are recommended after careful evaluation of these patients [32].

When suspicion is raised, a complete evaluation is necessary, as an electrocardiogram may be normal in many cases, and even cardiac echocardiography may be misleading, even in repeated evaluation [33]. An angiographic evaluation of the coronary arteries is mandatory to rule out any suspicion of the disease.

## 5. Conclusions

Diagnosis of ARCAPA is challenging, and repeated echocardiographic evaluation should be performed when a mild suspicion is raised. If the coronary arteries appear dilated then angiography of the coronary arteries, either invasive or by angioCT or angioMRI, should be recommended.

Although most of the patients with ARCAPA remain asymptomatic, reimplantation of the right coronary artery into the ascending aorta may be recommended to prevent myocardial hypoperfusion and sudden death or another complication. In association with other cardiac lesions, the initial echocardiographic evaluation may be misleading.

## Figures and Tables

**Table 1:** Diagnostic tools in ARCAPA.

Category	Characteristics
**Clinical**	− asymptomatic or − symptoms of exertion angina, dyspnea, heart failure, syncope
**ECG**	− normal or non-specific modifications: ○ deep Q waves in inferior leads; ○ negative T waves in lateral derivations; ○ ST elevation in acute coronary lesion; ○ left ventricular hypertrophy.
**Echocardiography**	− dilated left coronary artery; − normal RCA origin not detectable; − RCA origin from the pulmonary artery, shifted to the left, with retrograde, blue diastolic flow data.
**AngioCT, angioMRI**	− right coronary artery origin from the pulmonary artery; − dilated collateral circulation − evidence of fibrosis on gadolinium enhancement.
**Invasive** **coronarography**	− absent right coronary artery origin from the aorta; − dilated left coronary artery and branches; − abundant collateral circulation with retrograde filling of the right coronary artery; − right coronary artery connected to the pulmonary artery.

## Data Availability

Not applicable.
